# Super-Resolution Deep Learning Reconstruction Improves Image Quality of Dynamic Myocardial Computed Tomography Perfusion Imaging

**DOI:** 10.3390/tomography12010007

**Published:** 2026-01-07

**Authors:** Yusuke Kobayashi, Yuki Tanabe, Tomoro Morikawa, Kazuki Yoshida, Kentaro Ohara, Takaaki Hosokawa, Takanori Kouchi, Shota Nakano, Osamu Yamaguchi, Teruhito Kido

**Affiliations:** 1 Department of Radiology, Graduate School of Medicine, Ehime University, Toon 791-0295, Japan; 2 Department of Radiology, Ehime Prefectural Imabari Hospital, Imabari 794-0006, Japan; 3 Canon Medical Systems Corporation, Otawara 324-8550, Japan; 4 Department of Cardiology, Pulmonology, Hypertension, and Nephrology, Graduate School of Medicine, Ehime University, Toon 791-0295, Japan

**Keywords:** computed tomography, myocardial perfusion imaging, deep learning, super resolution, myocardial blood flow, variability

## Abstract

**Background/Objectives**: Super-resolution deep-learning reconstruction (SR-DLR) is an advanced image reconstruction technique, but its effect on dynamic myocardial computed tomography perfusion (CTP) imaging has not been evaluated. This study aimed to examine the impact of SR-DLR on image quality and perfusion parameters in dynamic myocardial CTP. **Methods**: Thirty-five patients who underwent dynamic myocardial CTP for coronary artery disease assessment were retrospectively analyzed. Two CTP datasets were reconstructed using hybrid iterative reconstruction (HIR) and SR-DLR. Image quality was compared qualitatively and quantitatively, including image noise, signal-to-noise ratio (SNR), contrast-to-noise ratio (CNR), and edge rise slope (ERS). Equivalence of CT-derived myocardial blood flow (CT-MBF) between two reconstructions was tested using a previously reported 15% equivalence margin. Intra-patient variability of CT-MBF was evaluated using the robust coefficient of variation (rCV). **Results**: In the qualitative assessment, SR-DLR had significantly higher scores in contrast (4.0 vs. 2.0) and sharpness (4.5 vs. 2.5) compared with HIR (*p* < 0.001), while contrast scores were similar. In the quantitative assessment, SR-DLR demonstrated significantly lower image noise (19.4 vs. 29.4 HU), and improved SNR (6.1 vs. 4.1), CNR (13.7 vs. 10.9), and ERS (171.0 vs. 135.1 HU/mm) (all *p* < 0.001). Mean global CT-MBF was comparable (3.15 ± 0.91 mL/g/min for HIR vs. 3.18 ± 0.97 mL/g/min for SR-DLR) and equivalence was confirmed (*p* = 0.022). SR-DLR significantly reduced rCV compared with HIR (36.0% vs. 41.0%, *p* < 0.001). **Conclusions**: SR-DLR enhances image quality in dynamic myocardial CTP while maintaining mean global CT-MBF and reducing intra-patient variability.

## Simple Summary

Super-resolution deep-learning reconstruction (SR-DLR) has been reported to improve image quality in computed tomography (CT) imaging, including the evaluation of coronary artery stenosis and the luminal assessment of coronary stents. However, its potential impact on dynamic myocardial CT perfusion (CTP) imaging has not been investigated. In our results, SR-DLR significantly improved the image quality of dynamic CTP images and reduced intra-patient CT-derived myocardial blood flow variability without altering the mean values. These findings suggest that SR-DLR has the potential to be applied to myocardial CTP imaging and may be useful for the assessment of CTP data.

## 1. Introduction

Myocardial computed tomography perfusion (CTP) imaging has been established as a technique for evaluating myocardial ischemia and is reported to be superior to single-photon emission computed tomography (SPECT) due to its higher spatial resolution [1,2]. Dynamic myocardial CTP acquires multiple consecutive data points during the first-pass transit of contrast medium through the myocardium under pharmacological stress, which enables the quantitative assessment of computed tomography-derived myocardial blood flow (CT-MBF) [3]. Quantitative assessment of myocardial perfusion using CT-MBF is more objective and reproducible than visual assessment, with high diagnostic performance for detecting myocardial ischemia [4,5,6].

However, dynamic myocardial CTP has a major limitation of comparatively high radiation exposure [5]. The low tube potential scan is a well-known technique for enhancing image contrast and reducing radiation dose in myocardial CTP imaging; however, it increases image noise [7]. Iterative reconstruction (IR) is widely adopted to suppress the elevated image noise encountered at low tube potentials; however, when the radiation dose is too aggressively curtailed, IR can introduce an undesirable over-smoothing artifact [8,9]. Recently, deep-learning image reconstruction (DLR) has been developed as a new technique that employs a deep convolutional neural network (DCNN) trained on high-quality reference data, which delivers higher subjective image quality than fully model-based IR, while reducing reconstruction time [9,10]. Moreover, super-resolution DLR (SR-DLR) has emerged as a novel image reconstruction technique generated by training a super-resolution DCNN with high-quality cardiac images obtained from ultra-high-resolution computed tomography (UHR-CT) [11]. SR-DLR can not only reduce image noise but also enhance spatial resolution compared to normal-resolution DLR (NR-DLR) [12]. Although SR-DLR has been used in clinical practice, including for the evaluation of coronary artery stenosis, luminal coronary artery stents, and delayed myocardial enhancement [13,14,15], its effect on dynamic myocardial CTP imaging remains unclear.

The novelty of this study lies in evaluating, to the best of our knowledge, the applicability of SR-DLR to dynamic myocardial CTP imaging for the first time. The main contribution of this study is to investigate the impact of SR-DLR on the image quality and quantification of CT-MBF in dynamic myocardial CTP imaging.

The remainder of this paper is organized as outlined below. Section 2 describes the study population, imaging protocol, image reconstruction, analysis methods and statistics. Section 3 presents the results of the qualitative and quantitative image quality analyses and CT-MBF quantitative analysis. Section 4 discusses the validity of these research findings, clinical implications and limitations. Section 5 concludes the paper and suggests future prospects.

## 2. Materials and Methods

### 2.1. Study Population

This retrospective study included 37 consecutive patients who underwent dynamic myocardial CTP for the assessment of coronary artery disease (CAD) between December 2017 and July 2018. The study was conducted in accordance with the Declaration of Helsinki and was approved by the Institutional Review Board of Ehime University Hospital (No. 2303016) on 20 March 2023.

### 2.2. Dynamic Myocardial CTP Scan Protocol and Post-Processing of Image Reconstruction

Dynamic myocardial CTP was performed using a previously established protocol employing a 320-detector row CT scanner (Aquilion ONE GENESIS Edition; Canon Medical Systems Inc., Otawara, Japan) with an automatic dual injector (Stellant DualFlow; Nihon Medrad KK, Osaka, Japan) [16]. To determine the arrival time of contrast medium, a timing bolus scan was conducted at the proximal ascending aorta using a 20% diluted iopamidol solution (370 mg iodine/mL, Bayer Yakuhin, Ltd., Osaka, Japan) infused at 5.0 mL/s for 10 s, followed by a saline flush for 4 s at the same rate. CTP data acquisition was initiated 6 s prior to the arrival of the contrast medium at the ascending aorta. After administering the adenosine triphosphate infusion (0.16 mg/kg/min) for 3 min, a dynamic myocardial CTP scan was performed with the prospective ECG-triggered scan mode at 45% RR interval and contrast medium (5 mL/s for 10 s), followed by a saline chaser (5.0 mL/s for 4 s). CTP datasets were acquired every other heartbeat until the completion of the first pass of the contrast medium through the myocardium. The scanning parameters were as follows: tube potential, 80 kVp; tube current, 300 mA; gantry rotation time, 0.275 s; detector configuration, 320 × 0.5 mm; effective coverage, 100 mm.

The image reconstruction was performed with a slice thickness of 1 mm using the following two methods.: (a) hybrid IR (HIR) (adaptive iterative dose reduction 3D: AIDR 3D, FC03, standard, Canon Medical Systems) and (b) SR-DLR (Precise IQ Engine: PIQE, Cardiac, standard, Canon Medical Systems).

### 2.3. Qualitative and Quantitative Image Analyses

All image quality was assessed using axial CTP images with 1-mm slice thickness and average intensity projection. A representative phase at the peak enhancement of the ascending aorta was selected from the dynamic CTP series.

Two experienced radiologists (with 12 and 16 years of experience in cardiac imaging, respectively) assessed qualitative image quality using a dedicated workstation (Ziostation2; Ziosoft Inc., Tokyo, Japan) under blinding to all clinical and reconstruction information. The radiologists independently evaluated axial images from the two different CTP datasets in random order, focusing on noise, contrast, and sharpness using a 5-point scale (1 = non-evaluable, 2 = fair evaluable, 3 = moderate image quality, 4 = good image quality, 5 = excellent image quality) under optimized window level/width settings for each case [16]. The visual score was determined by averaging the scores from both readers.

An experienced radiologist (with six years of experience in cardiac imaging) assessed the quantitative image quality using software (ImageJ, version 1.54p; National Institutes of Health, Washington, DC, USA) under blinding to all clinical and reconstruction information. Regions of interest (ROI) (12 mm in diameter) in the ascending aorta were used to measure standard deviation (SD), which was defined as image noise in this study. ROIs (8 mm in diameter) in the left ventricular (LV) septum and subcutaneous fat were carefully placed to measure the CT number and SD. The signal-to-noise ratio (SNR) and contrast-to-noise ratio (CNR) were calculated using the following equations: SNR = CT_septum_/SD_septum_; CNR = (CT_septum_ − CT_fat_)/SD_fat_. Additionally, a profile curve (10 mm in length) from the LV septum to the LV cavity was used to assess the edge-rise slope (ERS). ERS was assessed for image sharpness and defined as a slope between 10% and 90% of the CT number on the profile curve [17]. The image noise, SNR, CNR, and ERS were measured three times at random intervals, and the average value was compared between two CTP datasets with HIR and SR-DLR.

### 2.4. CT-Derived MBF Analysis

An experienced radiologist (with five years of experience in cardiac imaging) blindly performed quantification of CT-MBF using a dedicated workstation (Vitrea, Canon Medical Systems, Otawara, Japan). The global CT-MBF was calculated at the patient level for two dynamic CTP datasets using HIR and SR-DLR. The robust coefficient of variation (CV) for CT-MBF within the patient was calculated to evaluate intra-patient CT-MBF variability based on the median-nIQR method [18].

### 2.5. Statistical Analysis

Continuous data are expressed as the mean ± SD or as the median (25th percentile–75th percentile), depending on their distribution. The Shapiro–Wilk test was used to assess normality. Differences in qualitative and quantitative image quality scores were compared between the HIR and SR-DLR CTP datasets using the Wilcoxon signed-rank test. The equivalence of the mean global CT-MBF with SR-DLR was assessed using paired-sample two one-sided *t*-tests (TOST) referenced to that with HIR [19]. Equivalence is demonstrated when the two-sided 90% confidence interval for the paired mean difference lies entirely within the pre-specified margin of ±15% of the HIR mean. This margin is consistent with reported PET-MBF test–retest variability [20]. In addition, a previous study in patients without ischemia has reported intra-individual CT-MBF variability of approximately 85% to 113% [21], which suggests that a 15% margin is acceptable. For equivalence testing, a one-sided significance level of α = 0.05 was used. Robust CV for CT-MBF within patients was compared using the Wilcoxon signed-rank test. In all tests, statistical significance was set at *p* < 0.05. All statistical analyses were performed using the R software (Version 4.3.3, https://www.r-project.org).

## 3. Results

### 3.1. Study Population

**Figure 1:**
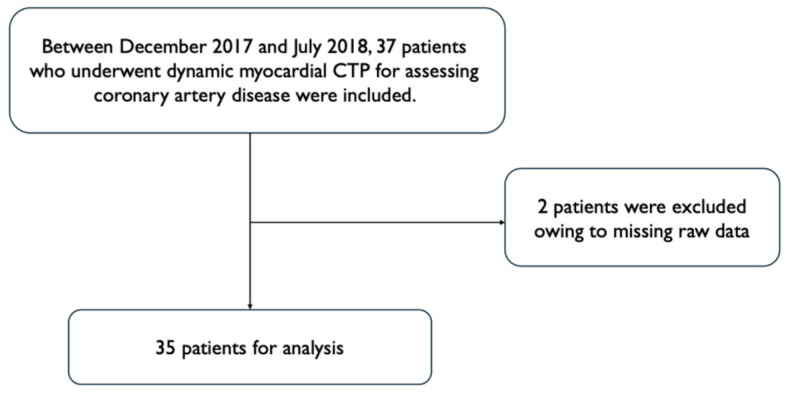
Flowchart of the patient selection (CTP, computed tomography perfusion).

Of the 37 patients, two were excluded due to missing raw data. Finally, 35 eligible patients were enrolled in the present study (Figure 1). The patient characteristics are summarized in Table 1. The median of volume CT dose index and dose-length product associated with dynamic myocardial CTP were 28.4 ± 5.5 mGy and 283.7 ± 55.3 mGy · cm, respectively.

### 3.2. Qualitative and Quantitative Image Quality

**Figure 2:**
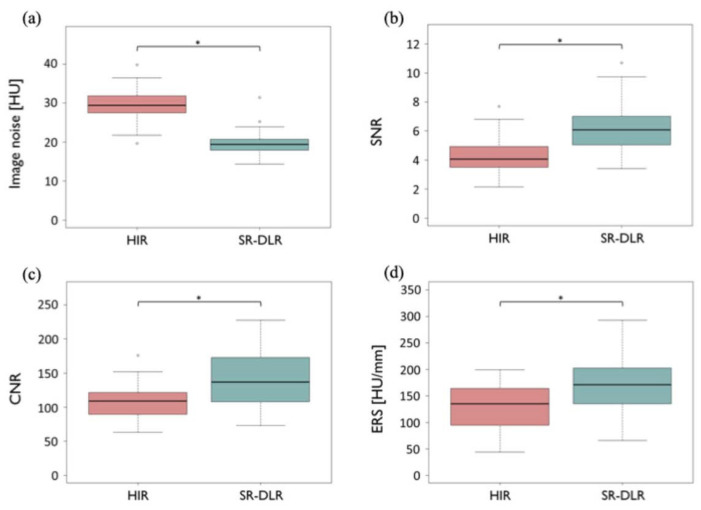
Box plots of hybrid iterative reconstruction and super-resolution deep learning reconstruction on quantitative image quality. (**a**) Image noise, (**b**) SNR, (**c**) CNR, and (**d**) ERS for comparison between HIR and SR-DLR. Image noise, SNR, CNR, and ERS significantly improved with SR-DLR compared to HIR. * *p* < 0.001. (CNR, contrast-to-noise ratio; ERS, edge rise slope; HIR, hybrid iterative reconstruction; HU, Hounsfield unit; SNR, signal-to-noise ratio; SR-DLR, super-resolution deep learning reconstruction).

The results of the qualitative and quantitative image quality assessments are presented in Table 2 and Table 3. For the qualitative image quality assessment, the scores for noise and sharpness with SR-DLR were significantly higher than those with HIR (*p* < 0.001), and no significant difference was observed in the scores for contrast between the two reconstruction methods (*p* = 0.053). For the quantitative image quality assessment, SR-DLR significantly improved image noise (*p* < 0.001), SNR (*p* < 0.001), and CNR (*p* < 0.001) compared to HIR (Figure 2a–c). Additionally, SR-DLR significantly enhanced ERS compared to HIR (*p* < 0.001) (Figure 2d).

### 3.3. Equivalences and Variations of Global CT-MBF

**Figure 3:**
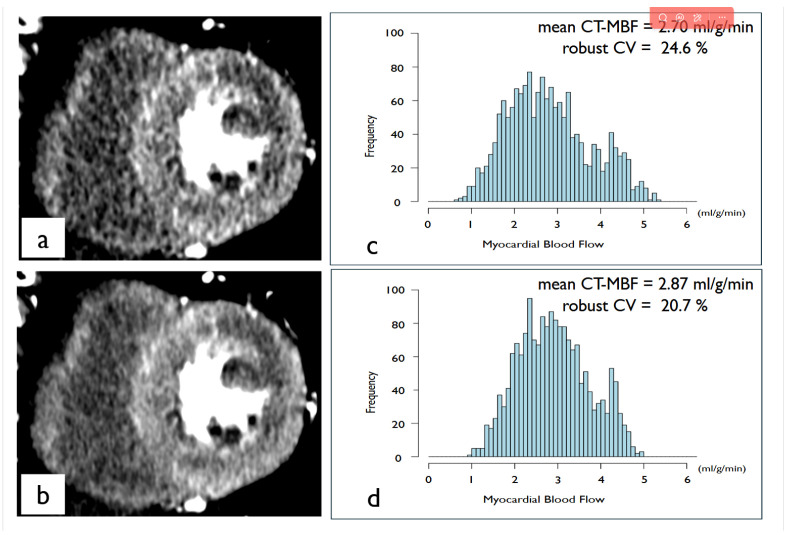
A representative case of super-resolution deep learning reconstruction applied to dynamic myocardial computed tomography perfusion imaging. Short-axis images reconstructed using HIR (**a**) and SR-DLR (**b**). Histograms of the CT-MBF with HIR (**c**) and SR-DLR (**d**). SR-DLR reduces image noise and enhances sharpness compared to HIR. In histogram analysis, mean CT-MBF remained largely unchanged by using SR-DLR, but SR-DLR reduced the intra-patient variability of CT-MBF, leading to a decreased robust CV. (CT-MBF, computed tomography-derived myocardial blood flow; CV, coefficient of variation).

The mean global CT-MBFs were 3.15 ± 0.91 mL/g/min for HIR and 3.18 ± 0.97 mL/g/min for SR-DLR. The mean global CT-MBF with SR-DLR was equivalent to that with HIR (*p* = 0.022). For the assessment of intra-patient CT-MBF variability, the robust CVs for CT-MBF within the patient were 41.0 (28.1–48.1) % for HIR and 36.0 (23.9–44.3) % for SR-DLR. The robust CV for CT-MBF within the patient was significantly reduced using SR-DLR compared to HIR (*p* < 0.001). A representative case is shown in Figure 3.

## 4. Discussion

The main findings of this study were as follows: (1) SR-DLR significantly improved the image quality of dynamic myocardial CTP imaging compared to HIR in both qualitative and quantitative assessments; (2) The mean global CT-MBF was significantly equivalent between the CTP images reconstructed with HIR and SR-DLR; (3) CT-MBF with SR-DLR showed significantly lower intra-patient variability compared to HIR.

HIR is an image-reconstruction framework that combines elements of filtered back-projection (FBP) and fully iterative algorithms, enabling markedly greater noise suppression than conventional FBP, while preserving reconstruction times that remain compatible with routine clinical workflow [10]. As dynamic myocardial CTP acquires multiple temporal phases, resulting in numerous images, HIR is a practical reconstruction technique for dynamic myocardial CTP imaging. NR-DLR is also an image reconstruction technique that leads to a higher noise reduction effect than HIR, along with a reasonable processing time. Takafuji et al. reported that NR-DLR can significantly improve the image quality of dynamic myocardial CTP images without altering the CT-MBF [22]. SR-DLR also has a capability of reducing image noise [12], and the present study indicated that SR-DLR could more effectively reduce image noise than HIR, leading to higher SNR and CNR in dynamic myocardial CTP images. In the image quality assessment of sharpness, the qualitative score and ERS with SR-DLR were significantly higher than those with HIR. We speculate that image sharpness was enhanced due to both greater noise reduction and improved spatial resolution by SR-DLR in the present study. Although UHR-CT can theoretically yield high image quality comparable to that obtained with SR-DLR, it is not suitable for dynamic myocardial CTP imaging because current UHR-CT systems employ an insufficiently wide detector to cover the whole heart. SR-DLR has the advantage of achieving dynamic myocardial CTP images with lower noise and higher spatial resolution without the need for dedicated CT equipment.

According to our results, there was significant equivalence in the mean global CT-MBF between the CTP images with HIR and SR-DLR. The CT-MBF analysis software used in the present study was originally developed using dynamic myocardial CTP images reconstructed using HIR, and it was validated using oxygen-15-labelled water PET [23]. Therefore, HIR is clinically appropriate to ensure the robustness of CT-MBF quantification by using this dedicated software. However, radiation dose reduction with HIR is limited because HIR has an inferior noise reduction capability compared to that of SR-DLR [12,24]. Dynamic myocardial CTP has the inherent problem of a relatively high radiation dose, and SR-DLR has the potential to overcome this limitation. Emoto et al. showed that SR-DLR enabled 50% radiation dose reduction associated with cardiac CT without sacrificing image quality using a cardiac phantom [25]. Similar to findings in the previous study using cardiac CT, SR-DLR may allow for reducing radiation dose associated with dynamic myocardial CTP imaging; however, simulation or phantom experiments were not performed in this study and the effect on radiation dose reduction remains unclear.

Our study showed that intra-patient MBF variability was significantly reduced using SR-DLR compared to HIR. We speculate that the image quality improvement of dynamic myocardial CTP images by SR-DLR might contribute to a reduction in intra-patient MBF variability. A previous study has reported that the image noise reduction is associated with decreased variability in CT-MBF, similar to our findings. The reduction in CT-MBF variability may be advantageous for determining the extent of myocardial ischemia, which is clinically important for assessing patients with CAD because a broader extent has been associated with poor prognosis [26,27]. Bhave et al. demonstrated that reduced variability in CT numbers using HIR led to decreased overlap between the histograms of ischemic and normal myocardium and improved diagnostic accuracy of myocardial ischemia [28]. Similarly, it can be assumed that reduced variability in CT-MBF using SR-DLR misclassification between normal and ischemic myocardium leading to more accurate assessment of myocardial ischemia extent in dynamic myocardial CTP imaging. Furthermore, SR-DLR is also expected to provide a precise visual assessment by reducing image noise and enhancing spatial resolution. Morikawa et al. demonstrated that SR-DLR significantly improved the accuracy of coronary calcium volume quantification compared to FBP in a phantom study [29]. Takafuji et al. showed that SR-DLR improved the reproducibility of myocardial infarction quantification compared to HIR and NR-DLR [15]. Thus, SR-DLR may enable a more accurate and reproducible assessment of the extent of myocardial ischemia than conventional image reconstruction methods. However, further studies are needed to determine its validity and clinical usefulness.

This study had several limitations. First, this was a single-center study with a relatively small sample size, and the SR-DLR algorithm used in our study was vendor-specific, which may limit the generalizability of our findings. Second, it remains to be determined how much radiation dose reduction using SR-DLR is clinically acceptable. Future investigations including low-dose image-quality assessments using simulation or phantom experiments are needed to demonstrate the radiation dose reduction of SR-DLR. Third, the diagnostic and prognostic performance of SR-DLR for myocardial ischemia could not be assessed in the present study due to the small number of cases and the lack of a validation test for identifying myocardial ischemia. Future prospective multicenter studies are required to evaluate the clinical value of SR-DLR in patients with CAD.

## 5. Conclusions

In conclusion, SR-DLR significantly improved the qualitative and quantitative image quality of dynamic myocardial CTP images compared to HIR. Importantly, these image-quality improvements were achieved while preserving the mean global MBF. Furthermore, SR-DLR reduced intra-patient variability of MBF.

In this study, the capability of SR-DLR for reducing the radiation dose in dynamic myocardial CTP imaging was not evaluated. Additionally, the capability of SR-DLR for identifying myocardial ischemia was not investigated because of the small sample size with validation test for the definition of normal and ischemic myocardium. In the future, phantom studies and clinical studies with larger cohorts are needed to clarify the potential of SR-DLR for radiation dose reduction and for improving the assessment of myocardial ischemia in dynamic myocardial CTP imaging.

## Figures and Tables

**Table 1:** Patient characteristics.

Characteristics	
Age (years)	68.5 ± 10.1
Sex, male (% of total)	23 (66%)
Body mass index (kg/m^2^)	24.2 ± 3.0
Cardiovascular risk factors	
Hypertension	18 (51%)
Dyslipidemia	19 (54%)
Diabetes mellitus	10 (29%)
Smoking habit	12 (34%)
Family history of CAD	10 (29%)

Data are expressed as “mean ± SD” or “number (%).” CAD, coronary artery disease; SD, standard deviation.

**Table 2:** Qualitative image quality assessment.

	HIR	SR-DLR	* *p*-Value
Noise	2.0 (1.75–2.75)	4.0 (3.5–4.75)	<0.001
Contrast	4.0 (3.75–4.5)	4.0 (4.0–4.5)	0.0533
Sharpness	2.5 (2.0–3.0)	4.5 (4.0–5.0)	<0.001

Data are expressed as median (25th percentile–75th percentile). * Statistical significance was determined at *p* < 0.05 between the two CT datasets. (HIR, hybrid iterative reconstruction; SR-DLR, super-resolution deep-learning reconstruction).

**Table 3:** Quantitative image quality assessment.

	HIR	SR-DLR	* *p*-Value
Image noise (HU)	29.4 (27.5–31.9)	19.4 (17.9–20.8)	<0.001
SNR	4.1 (3.5–5.0)	6.1 (5.1–7.1)	<0.001
CNR	10.9 (8.9–12.2)	13.7 (10.7–17.3)	<0.001
ERS (HU/mm)	135.1 (94.1–164.0)	171.0 (129.7–203.8)	<0.001

Data are expressed as median (25th percentile–75th percentile). * Statistical significance was determined at *p* < 0.05 between the two CT datasets. (CNR, contrast-to-noise ratio; ERS, edge rise slope; HIR, hybrid iterative reconstruction; HU, Hounsfield unit; SNR, signal-to-noise ratio; SR-DLR, super-resolution deep learning reconstruction).

## Data Availability

The data used in this study cannot be shared publicly due to privacy concerns for the individuals who participated in the study. The data will be shared upon reasonable request from the corresponding authors.

## Abbreviations

CNR	Contrast-To-Noise Ratio
CT	Computed Tomography
CT-MBF	Computed Tomography-Derived Myocardial Blood Flow
CTP	Computed Tomography Perfusion
DCNN	Deep Convolutional Neural Network
DLR	Deep-Learning Image Reconstruction
ERS	Edge Rise Slope
HIR	Hybrid Iterative Reconstruction
IR	Iterative Reconstruction
NR-DLR	Normal-Resolution Deep-Learning Reconstruction
rCV	Robust Coefficient of Variation
SNR	Signal-To-Noise Ratio
SPECT	Single-Photon Emission Computed Tomography
SR-DLR	Super-Resolution Deep-Learning Reconstruction
UHR-CT	Ultra-High-Resolution Computed Tomography
